# Relations between Effects and Structure of Small Bicyclic Molecules on the Complex Model System *Saccharomyces cerevisiae*

**DOI:** 10.3389/fphar.2017.00170

**Published:** 2017-03-30

**Authors:** Matteo Brilli, Andrea Trabocchi, Tobias Weil, Duccio Cavalieri, Irene Stefanini

**Affiliations:** ^1^Centre for Research and Innovation, Fondazione Edmund MachTrento, Italy; ^2^Department of Chemistry “Ugo Schiff”, University of FlorenceFlorence, Italy; ^3^Department of Biology, University of FlorenceFlorence, Italy; ^4^Division of Biomedical Cell Biology, Warwick Medical School, University of WarwickCoventry, UK

**Keywords:** small compounds, *Saccharomyces cerevisiae*, phenotypic screening, principal component analysis, stepwise regression analysis, drug screening, drug development, high-throughput screening (HTS)

## Abstract

The development of compounds able to modify biological functions largely took advantage of parallel synthesis to generate a broad chemical variance of compounds to be tested for the desired effect(s). The budding yeast *Saccharomyces cerevisiae* is a model for pharmacological studies since a long time as it represents a relatively simple system to explore the relations among chemical variance and bioactivity. To identify relations between the chemical features of the molecules and their activity, we delved into the effects of a library of small compounds on the viability of a set of *S. cerevisiae* strains. Thanks to the high degree of chemical diversity of the tested compounds and to the measured effect on the yeast growth rate, we were able to scale-down the chemical library and to gain information on the most effective structures at the substituent level. Our results represent a valuable source for the selection, rational design, and optimization of bioactive compounds.

## Introduction

Drug discovery strongly relies on the availability of small molecule compounds bearing biological activity (Smietana et al., 2016). During the last decades, several approaches have been adopted aiming at the exploration and exploitation of the chemical variability (Richard et al., 2008). Among these, a noteworthy contribution was given by Diversity-Oriented Synthesis (DOS), which allowed the generation of the widest diversity and complexity from a simple initial scaffold (O'Connell et al., 2013). Large libraries generated with a DOS approach are a valuable resource to explore the effect of small structural changes on the biological activity of a compound (Bajorath, 2013). Once a large set of compounds is synthesized, an efficient and high-throughput assay is necessary to assess the library bioactivity (Ghannoum and Rice, 1999; Huang et al., 2004). Both target-oriented and chemical genetics-oriented approaches have been adopted to rapidly and efficiently test the biological activity of large sets of compounds (Cong et al., 2012). While target-oriented screenings offer the advantage of an *a priori* knowledge of the entity affected by the tested compound, chemical genetics approaches show other advantages. As an example, cell- or organism- based screenings allow evaluating the biological function of the tested compounds without the pre-selection of a specific target, hence allowing the discovery of new therapeutic targets (Schenone et al., 2013). For the same reason, chemical genetics is useful for discovering compounds active against “undruggable diseases,” associated with complex interactions of different biological entities (i.e., protein-protein, protein-DNA), for which targeted-oriented approaches can not be adopted (Altmann et al., 2009). Furthermore, the cellular context of chemical genetics allows to assessing whether the efficacy of the compound under study is reduced by metabolic reactions or by physical cellular barriers (i.e., the cell membrane) (Swinney and Anthony, 2011). All these advantages made chemical genetics the most successful approach for evaluating the activity of small-molecule libraries (Swinney, 2013). The downside of such phenotypic screenings is that they allow the identification of active compounds, but further studies are necessary afterward to identify their targets (Schenone et al., 2013).

The budding yeast *Saccharomyces cerevisiae* is mainly used for wine, beer, and bread fermentations but is also suitable for high-throughput screening approaches (Schenone et al., 2013). In fact, it has been successfully used as a model organism in phenotypic screens aimed at the identification of molecules of pharmacological interest (Brenner, 2004; Hoon et al., 2008). Among the others, a matter of clinical relevance could largely benefit from the testing of parallel synthesis product on the budding yeast: systemic fungal infections, which have been estimated to cause more casualties than malaria (http://www.bbc.co.uk/news/health-36702215). Because of the similarity between *S. cerevisiae* and pathogenic fungi, compounds inhibiting the growth of the former are also potentially active against *A. fumigatus, C. albicans* and other important human pathogens (Goldstein and McCusker, 2001). By testing the chemical variability offered by DOS libraries on *Saccharomyces cerevisiae* it is therefore possible to assess the relationship between chemical variance and biological activity and get insights about much more complicated systems.

Here we dissected the relations between the *S. cerevisiae* phenotypic growth effects and chemical structures of 101 small bicyclic compounds. The molecules object of this study were generated by combining different functional groups with three scaffolds: BTAa (Bicycles from Tartaric and Amino acids), BTK (Bicycles from Tartaric and amino ketones) and BTG (Bicycles from Tartaric acid and Glyceraldehyde derivatives). In a previous study from our group (Stefanini et al., 2010), we evaluated the effect of these compounds on the growth rate of several *S. cerevisiae* strains. This study allowed the selection of promising antifungal compounds, one of which (named 089) was the most effective in reducing the yeast growth. In the present study, we took advantage of the high degree of chemical diversity of the tested compounds and the measured effects on the yeast growth rate to scale-down our chemical library and to gain information on the most effective (combinations of) substituents providing a valuable source for the rational design of bioactive compounds and their optimization.

## Materials and methods

### Compounds

The tested library consisted of BTAa (Bicycles from Tartaric acid and Amino acids), BTK (Bicycles from Tartaric acid and amminic ketones) and BTG (Bicycles from tartaric acid and glyceraldehyde derivatives) compounds (Trabocchi et al., 2008). BTAa were synthesized from 3-aza-6,8-dioxa-bicycle[3.2.1]octane as the fundamental structure and variously functionalized as previously described (Machetti et al., 2007). Stability of the ketal moiety over strong aqueous acid conditions has been assessed by several applications of these compounds in peptide synthesis. For an account of all the chemical features of these compounds, please refer to Trabocchi et al. (2006). For uniformity, all molecules were dissolved in DMSO at the same stock concentration (5 mM) and were compared to the same control (DMSO in volumes equal to the volume of the treatment molecule). Molecule structures are shown in Supplementary Figure 1.

### Yeast strains, culturing and measurement of phenotypic effects

The *Saccharomyces cerevisiae* strains used in this study are: the wild type strains BY4742 and W303, and three BY4742 deletion strains, Δerg6, Δpdr3, and Δsnq2. Yeast cells were grown in liquid YPD medium (2%Yeast extract, 1% peptone, 2% Dextrose) at 27°C with orbital shaking. Before treatment, yeast cells were pre-cultured overnight. Cells were then inoculated at 5^*^10ˆ5 cells/ml in liquid YPD supplemented with either the molecule (0.3 mM) or DMSO (equal volumes, see details in the previous section). The effects induced by molecules on *S. cerevisiae* growth were summarized as the difference of the Optical Density at the stationary phase (ODst) of the treated culture compared to the control (treated with DMSO) and expressed as a percentage of the ODst of the untreated culture. The ODst percentages calculated for each molecule on the tested strains are listed in Supplementary Table 1.

### Principal component analysis

Principal component analysis (PCA) was carried out by using as cases the 19 molecules selected as reducing the ODst of the wild-type *S. cerevisiae* W303 strain and as descriptors (variables) both the chemical characteristics (substituents at the R1, R2, R3, R4, and R4″ positions and molecule scaffolds) and the phenotypic effects (ODst %) on tested strains. Since variables encompassed both qualitative (chemical groups) and quantitative (phenotypic effects) descriptors, we used the dudi.mix function of the R package ade4 (Dray and Dufour, 2007), combining PCA for the quantitative factors and MCA (Multiple Correspondence Analysis) for qualitative ones (see ade4 R package documentations for further details, Dray and Dufour, 2007).

### Redundancy analysis

To study the relevance of the molecules' general features (other than chemical substituents) on the biological effect, we explored the relationship of chemical hindrance and polarization with the phenotypic effects of the molecule. We performed redundancy analysis (RDA, rda function of the vegan R package, Oksanen et al., 2016) to examine how much variation in molecular features explain the variation in the phenotypic effect. In particular, RDA was carried out on two separate matrices, one including the phenotypic effects of the 19 selected molecules on the complete set of strains, the other including the chemical features (either the polarizability or the hindrance calculated for the whole molecule or the substituents separately). Molecules and chemical moieties polarizabilities were calculated using the Marvin Sketch software (5.10.0) and the tendency of the analyzed entity to diminishing the external electric field was calculated with a method based on the Thole's parameters (Jensen et al., 2002). Similarly, molecules and substituents steric hindrances were calculated by using the Marvin Sketch software (5.10.0) and evaluating the van der Waals volume of the conformer (in Armstrong3) (Jensen et al., 2002). Molecular and substituents hindrance and polarizability values are listed in Supplementary Table 1.

### Stepwise regression analysis

Stepwise regression analysis was carried out to identify the chemical variables (substituents or scaffolds) which are most responsible for the biological effect observed in experiments. Molecules functionalizations and scaffolds were translated into dummy variables indicating their presence or absence. Stepwise regression analysis was carried out on molecules inducing an ODst >2% or < −2% on the wild-type W303 strain compared to the ODst of the control culture using the stepAIC function of the MASS package (Venables and Ripley, 2002). Stepwise regression analysis consisted in the assessment of linearity among the biological effect (ODst on W303 strain) and the chemical structure tested by adding or removing one-by-one the variables and evaluating whether the change improved the model. Briefly, the dummy variables indicating the presence/absence of substituents and scaffolds were grouped according to their position (in case of substituents) or type (for scaffolds). Because stepwise regression analysis works by adding or removing one variable per step, the most complex model encompassed one dummy variable for each group, and the most simple model encompassed a dummy variable for one group only. Each modification of the variable set used to generate the model represented a single step. For each step, the resulting model was evaluated using the AIC (Akaike Information Criterion). The best model was considered the one with the highest AIC. The substituents that appeared only once in the chemical library were removed from the dataset to avoid the generation of misleading models.

## Results

### Evaluation of the biological activity of the chemical library

**Figure 1:**
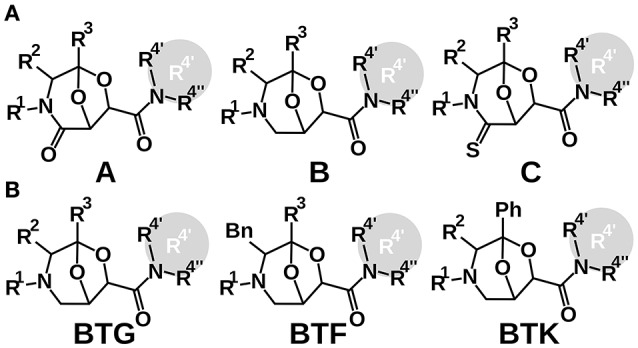
**Two classifications of the scaffolds the chemical library is built on. (A)** BTA(O), BTA and BTA(S); **(B)** BTK, BTF, and BTG. When a compound bore a cyclic residue at position R4′ and R4″, it was annotated as R4′.

The chemical diversity of the 101 analyzed compounds (molecular formula in Molecular_formula_strings.csv, drawings in Supplementary Figure 1) stems from three BTAa (Bicycles from Tartaric acid and Amino acids) scaffolds, combined with different R1, R2, R3, and R4′-4″ appendages (Figure 1) (Stefanini et al., 2010). All BTAas are derived from tartaric acid and one amino acid (Guarna et al., 1999; Trabocchi et al., 2002, 2003; Cini et al., 2006; Machetti et al., 2007). BTAas can be classified in two ways: according to the absence/presence of a double bonded Oxygen or Sulfur (Scaffold B, A, and C, Figure 1A) (Guarna et al., 1999), or according to the chemical reactants used to synthesize them (Figure 1B) (Machetti et al., 2007).

In our previous study (Stefanini et al., 2010), we observed that only 30 of the 101 compounds composing the tested library reduced the Optical Density at the stationary phase (ODst) of the wild-type *Saccharomyces cerevisiae* W303 strain when tested at a 0.3 mM concentration. To explore the drug potential of the tested compounds against the widest range of fungal and mammalian cells, it is pivotal to elude defenses specific of the organism used as a model to test the molecule bioactivity. Aiming at this, the selected compounds were further tested on a set of strains deleted in genes known to participate in an accurate and advanced response to drugs that characterizes *S. cerevisiae* and a few other fungi (Wolfger et al., 2001). In particular, the activity of the selected compounds was evaluated in strains deleted in either ERG6, SNQ2, or PDR3 genes and in the corresponding wild-type BY4742 strain as a reference. In our previous study, this round of phenotypic assays allowed us to identify, among the 30 molecules most active in the W303 strain, 19 compounds decreasing the ODst of the test strains by more than 20%, hence representing good candidates as fungicides (Stefanini et al., 2010).

### Identifying the relations between molecules' chemical variability and susceptibility to yeast defenses

**Figure 2:**
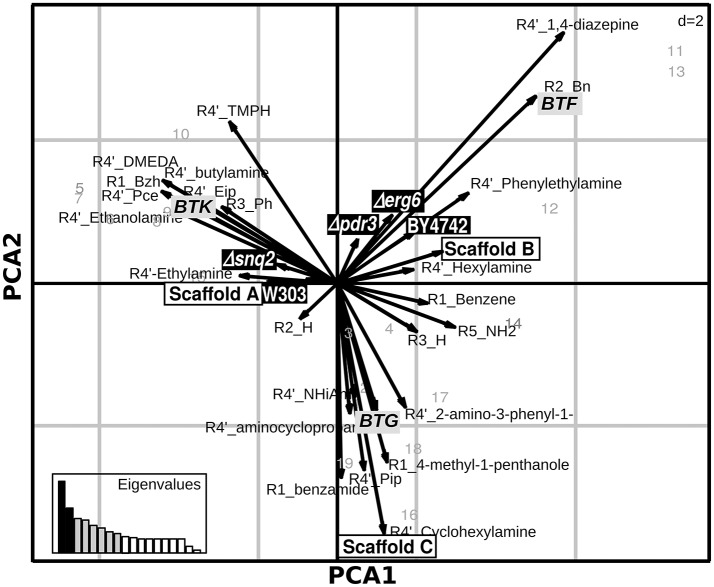
**Relationship among chemical features of selected compounds and their activity**. Duality diagram for the first two principal components obtained by analyzing correspondence between the ΔODst %and the chemical features of the molecules selected at the first-level. The cases for analysis were the molecules selected in the first level assay as able to induce a W303 ODst decrease higher than the 20% with respect to the ODst of the untreated culture. The variables are both the ΔODst % induced by the molecules on the tested strains and the molecule chemical features (substituent types, scaffold types); black-boxes labels: effects on the tested strains, white-boxes labels: molecules classification as shown in Figure 1A, gray-boxes labels: molecules classification as shown in Figure 1B. PCA1, First Principal Component; PCA2, Second Principal Component.

**Figure 3:**
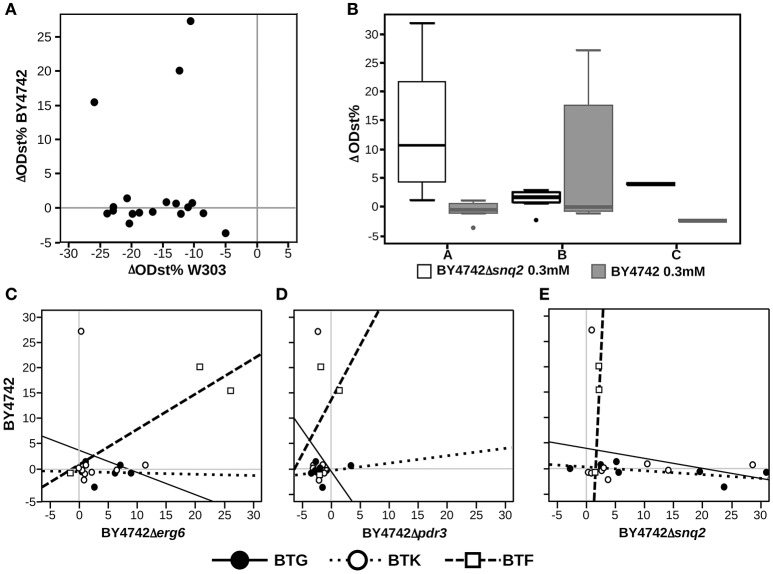
**Highlights of the effects of the selected molecules and their chemical characteristics. (A)** Direct comparison of the molecules effect on BY4742 and W303 strains, each dot indicate a different molecule; **(B)** boxplot showing the effects on the BY4742Δsnq2 strain culture ODst caused by the molecules grouped according to the scaffold classification showed in Figure 1A; **(C–E)** relationship between the effects induced by the molecules classified accordingly to Figure 1B on the wild-type BY4742 and on the Δerg6, Δpdr3, and Δsnq2 strains, respectively.

Exploring the relations between presence/absence of specific substituents or scaffolds and the effects induced on the set of deletion strains is helpful to understand which chemical features make the compound unaffectable by the yeast response to drugs. Aiming at this, we applied Principal Component Analysis (PCA) on the effects of the 19 molecules tested on the complete set of *S. cerevisiae* strains to explore the relations among chemical entities and the effects on the different strains (Figure 2). This analysis, carried out combining PCA for quantitative variables (the ODst values) and MCA (Multiple Correspondence Analysis) for qualitative variables (the scaffold and the R substituent at different positions), allows to exploring associations among variables measured for the library compounds (cases). The molecules inducing the highest ODst reduction on the W303 wild-type strain also proved to induce opposite effects on the deletion Δerg6 and Δpdr3 strains (as indicated by the corresponding arrows pointing to opposite directions in Figure 2). The effects induced on strains BY4742, Δerg6 and Δpdr3 were strongly correlated with the BTF class of compounds, while the other two classes of compounds were not correlated with any of the tested strains (Figure 2). In general, the BY4742 strain was less affected by the treatments than W303 (Figure 3A). The presence of either 1,4-diazepine or phenylethylamine at position R4″ and of benzene at R2, mainly associated with the BTF class, was correlated with the effect induced on the deletion strains Δerg6 and Δpdr3 and on the wild-type BY4742 strains (Figure 2). The presence of ethylamine at position R4″ was correlated to the effects induced on the wild-type strain W303 and on the deletion strain Δsnq2 (Figure 2). The effects induced on the Δsnq2 strain by the compounds based on scaffold A were more variable compared to those induced on other strains, comprising both different levels of inhibition and increase of yeast growth (Levene test, *P* = 0.005). Yet they showed an overall trend toward being more beneficial than all other compounds (Levene test, *P* = 0.005) as they induced a larger increase of culture ODst when compared to the control or to molecules of the B scaffold (Figure 3B). On the contrary, compounds built around scaffold B induced variable and beneficial effects on the wild-type BY4742 strain (Levene test, *P* = 0.025, Figure 3B). Different scenarios were observed when considering the effects induced by the selected molecules classified in terms of their scaffolds classified as BTG, BTF, or BTK (Figures 3C–E). Here, only BTF compounds induced proportional effects on both the Δerg6 and the BY4742 wild-type strains, while BTKs just had a slight effect on the growth of the BY4742 strain, and BTG molecules induced opposite effects on the wild-type and on the deletion strains -weak ODst decrease in the first and ODst increase in the latter- (Figure 3A). No other significant differences were observed when comparing the effects of the molecules belonging to the A, B, or C classes (Supplementary Figure 2) nor among the BTG, BTK, or BTF molecules (Supplementary Figure 3).

**Figure 4:**
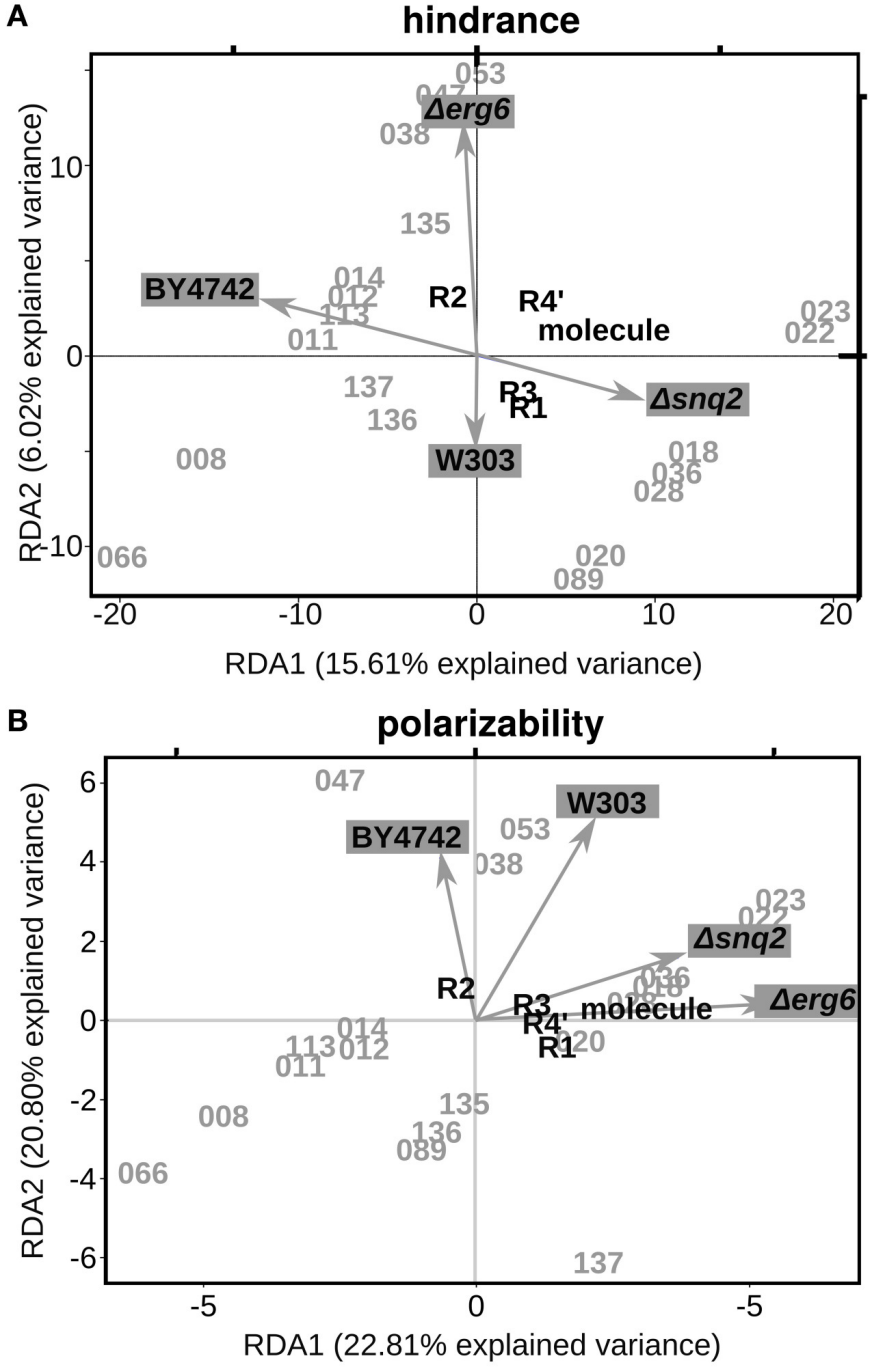
**Relationship between steric hindrance or polarizability and the phenotypic effects. (A)** First two explanatory components identified with the Redundancy Analysis (RDA) on ΔODst % values and steric hindrances. RDA1, First RDA constrained axis; RDA2, Second RDA constrained axis. The ΔODst % calculated for every strain treatment were used as constraining variables; as explanatory variables we used the steric hindrance values (A3) calculated with the Marvin Sketch software (5.10.0). **(B)** First two explanatory components identified with RDA on ΔODst % values and molecule/substituent polarizability. The ΔODst % calculated for every strain treatment were used as constraining variables; as explanatory variables we used the polarizability indexes (A3) calculated with the Marvin Sketch software (5.10.0).

Since steric hindrance and polarizability of the molecule (or parts of it) play a pivotal role in the compound's druggability (the ability of the drug to bind its target and exploit its activity), we explored the existence of a relationship among these two variables and the biological effects induced on the different strains. Aiming at this, we applied Redundancy Analysis to explore how much variation in molecular features (hindrance and polarizability) explains the variation in phenotypic effects. Briefly, RDA estimates the degree to which a block of variables can predict a second set of variables by linear relationships (Varmuza et al., 2012). In our study the two “blocks of variables” were the phenotypic measures (ODst for each selected molecule in all the tested strains) and the chemical features (either the polarizability or the hindrance calculated for the whole molecule or the substituents separately). Both the hindrance and the polarizability of the group at position R2 resulted to be important for molecules activity (Figure 4). The higher the R2 hindrance, the more pronounced was the positive effect on Δerg6 cell growth (Figure 4A, Supplementary Figure 4A). The molecule with the highest R2 polarizability (047) is also the molecule inducing the most beneficial effect on wild-type cells (Figure 4B, Supplementary Figure 4B). The level of polarizability of the complete molecule was related to the activity on strain Δerg6 (Figure 4B). Indeed, the molecule less prone to polarization (066) induced the highest cell death on the Δerg6 strain (Figure 4B). On the contrary, mild molecule polarizability (053 and 038) was associated to the highest beneficial effect on the Δerg6 strain (Supplementary Figure 4C). The same profile was shown by the relation between the hindrance of the group at position R4″ and the molecules effect on strain Δerg6 (Figure 4A). The molecule with the smallest R4″ group hindrance (066) was the one inducing the most deleterious effect on both Δerg6 and Δsnq2 strains, while the molecules with intermediate R4″ hindrance (053 and 038) were the most beneficial on the Δerg6 strain (Supplementary Figure 4D).

### Analysis of the complete chemical variance

**Figure 5:**
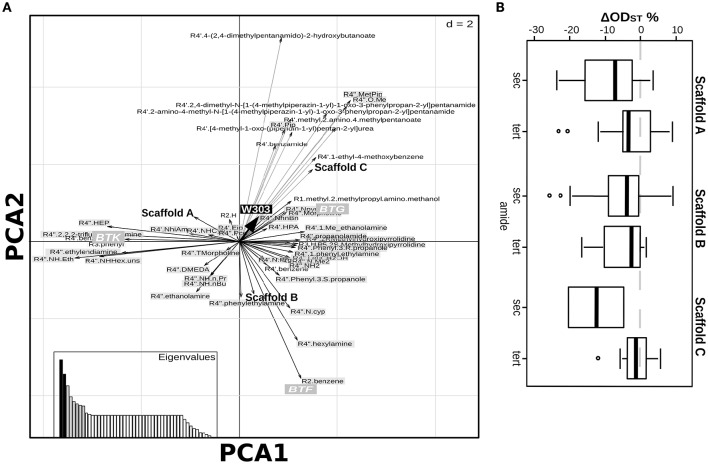
**Results of the analysis on the complete library of molecules. (A)** Duality diagram for the first two principal components obtained by analyzing the ΔODst % and the chemical features of the complete library of molecules. The cases for analysis were the molecules composing the library. The variables are the ΔODst % induced by the molecules on the W303 strain and the molecule chemical features (substituent types, scaffold types); black-box labels: effects on the tested strains, bold labels: molecules classification as shown in Figure 1A, gray-box labels: molecules classification as shown in Figure 1B. **(B)** Boxplots representing the influence of the presence of a secondary or tertiary amine in position R4″ in the effects induced on the W303 wild-type strain ODst by the molecules classified in three groups as described in Figure 1A.

The lesson learned from the effects on deletion strains of a selected pool of molecules gave several indications to dissect the contribution of the chemical functions to the biological effects the molecules can induce when the *S. cerevisiae*-specific response mechanisms are impaired. We went further, aiming at the exploration of the relationship between the chemical variance disclosed by the complete library and the biological effects induced on the wild-type W303 strain. The PCA on the ΔODst induced by the complete set of molecules revealed only weak correlations with some chemical features of the molecules (Figure 5A). A positive effect on the cell culture growth was associated to BTG molecules, to the C scaffold and to several chemical groups at positions R1 and R4′ (the corresponding arrows pointed in the same direction of the “W303” arrow, indicating the ODst variable, Figure 5A). On the contrary, neither the chemical groups at position R2 nor R3 were associated to any particular effect on the strain growth. The W303 ODst decrease (the opposite direction of the thick black arrow in Figure 5A) was not associated to any scaffold or BTA type, but it was weakly associated to the presence of n-propyl, n-Butyl, TMPH (thiomorpholine), DMEDA ((2-aminoethyl)dimethylamine) or ethanolamine at position R4′ or R4″. As regarding molecule functionalization, none of the chemical groups *per se* showed to be significantly correlated to the ODst variation (Wilcoxon test Supplementary Tables 2–4). A closer look into the PCA ordinations based on the results obtained from the complete set of molecules (Figure 5A) highlighted a trend related to the type of amide present at R4″ position. Indeed, a secondary amide at R4″ decreased the ODst with respect to the presence of a tertiary amide. In particular, the largest difference was evident for C scaffold molecules (BTA(S), Figure 1A), which reduced the ODst when bearing a secondary amide, whereas only slightly varied the ODst when a tertiary amide was present (Figure 5B, Wilcoxon test, *P* = 0.067).

**Figure 6:**
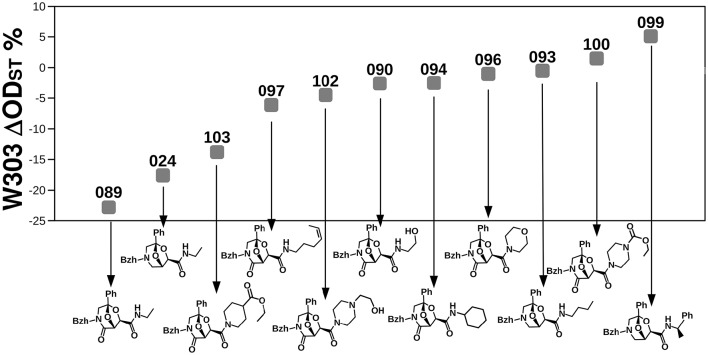
**Comparison of the effects induced on the W303 wild-type by the most fungicide molecule with respect to molecules almost identical to it but for small chemical features**. Numbers correspond to compound names.

The biological activity of a compound may be due to either single chemical groups or a combination of them. The identification of relationships between the biological activity and the combinations of chemical groups is instrumental to infer and design compounds inducing the desired fungicide/fungistatic effect. We thus carried out a series of stepwise regressions (19,440 complete models in total) by exploring every possible combination of scaffold types and chemical groups at the 4 positions (avoiding the combination of more than one substituent at the same position, see materials and methods). In other words, linearity among the biological effect and the chemical structure was tested by adding or removing one-by-one the variables and evaluating whether the change improved the model as summarized by the AIC (Akaike Information Criterion, a measure of the relative quality of statistical models). Because the linear model would be strongly affected by near-to-zero ODst, we carried out the analysis on the compounds inducing an ODst >2% or < −2% (71 compounds, Supplementary Table 1). Several combinations of chemical variables gave rise to small AIC (gray in Supplementary Table 5). All of them contained scaffold A (Figure 1A), and ethylamine at R4″ (gray in Supplementary Table 5); the detailed observation of the stepwise regression analysis showed that both of these molecular characteristics induced a decrease in W303 ODst (negative coefficient, Supplementary Table 6). In addition, the growth inhibition was also correlated with the presence of benzene at the R2 position (Supplementary Table 6, Supplementary Figure 5). Neither the molecule type (BTG, BTK, or BTF, Figure 1B) nor the type of substituent at R1 or R3 had a significant influence on the ODst. The compound showing the relevant chemical features revealed by the means of stepwise regression analysis (compound 089) resulted to be also the strongest growth inhibitor among the 19 molecules selected in the previous study after extensive experimental characterizations. This compound is a BTK build on the A scaffold with benzhydryl at position R1, a CH2 moiety at position R2, a phenyl at position R3 and an ethylamine at position R4″. The comparison of the effects on the wild-type W303 strain induced by molecules identical to 089 for all but one chemical group revealed interesting information (Figure 6). The substitution of the scaffold (024, scaffold A) reduced only slightly the activity of the compound (from −22.8% to −17.60%). Instead, the substitution of the R4″ ethylamine with other chemical groups reduced the activity of the compound from −22.8 to −13.8% (089 and 103, respectively), or even induced the opposite effect (100 and 099 are associated to a 1.5 and 5.1% ODst, respectively) (Figure 6).

## Discussion

The present analysis has been carried out on an entire organism, a complex system, thus the full dissection of the contribution of the chemical functions to the effects was highly complex too. Nevertheless, the combination of biological and chemical variances allowed us to draw general indications. The present study was not aimed at identifying the chemical features optimizing a specific mechanism of action. Rather, this organism-level analysis revealed valuable information that can be used to ameliorate the molecule chemical structure aiming at the optimization of the effect on the growth rate of *S. cerevisiae*. Irrespective of the drug chemical structure, the W303 strain was more sensitive than BY4742 to the treatment with BTAa compounds. These two wild-type strains mainly differ for the presence in BY4742 of a malfunctioning HAP1 gene reducing the BY4741 respiration. The higher sensitivity of W303 may therefore suggest that respiratory metabolism is involved in the pathway targeted by these molecules. By exploring the relations among structures and phenotypes for only the compounds selected as inducing the greatest effect on the wild-type W303 strain, we obtained objective information, which could not be summarized by visually inspecting the phenotypic results and the chemical structures. The classification of the tested molecules into three groups based on the absence/presence of a double bonded Oxygen or Sulfur (Scaffold B, A, and C, respectively) allowed to uncover the backbones' potential. While the presence of a sulfur atom on the principal ring [BTA(S), scaffold C, Figure 1A] made the molecule more prone in enhancing growth, two different (but still unknown) mechanisms of action can be hypothesized for the BTA(O) (scaffold A) and BTA (scaffold B). Molecules based on scaffold A generate a benefit to the cell, but this effect is avoided by their extrusion by the cell due to the Snq2 protein. On the contrary, wild type cells benefit from molecules built on scaffold B (BTA), but the this effect is not observed when Snq2p is missing. This could indicate that the molecules built on scaffold B influence cell metabolism by increasing respiration. Indeed, Snq2p requires ATP to extrude the drugs, which may explain the respiration request (Dueñas-Sánchez et al., 2010). *S. cerevisiae* can both breathe and ferment, but it has been shown that respiration increases biomass production (Decottignies et al., 1995). Thus, by producing more ATP and consuming more oxygen, the biomass increases, similar to the induction observed during the treatment of the wild type cells with the molecules build on the B scaffold. On the contrary, the lack of the Snq2 protein reduces ATP request, thus reducing the biomass production and restoring the situation of the untreated control upon the treatment with compounds bearing the B scaffold. A different story can be depicted when comparing molecules classified as BTF, BTK, and BTG. The BTG class of molecules (generated by the combination of tartaric acid and glyceraldehyde derivatives) was the only one not modifying the ODst of the wild-type BY4741 but increasing the ODst of the deletion strains. This result clearly indicates that the (beneficial) activity of these molecules in the BY4741 genetic background is affected by the yeast response/resistance to drugs. Indeed, cell growth is improved by this class of molecules only when the cell lacks its defense systems. Yet, the molecules bearing the phenyl group at R3 (BTKs) were the less affected by the yeast drug response, and induced effects having the same trend on both deletion and wild-type strains. The known drug response machinery does not affect the activity of these molecules. Several characteristics of the molecules, in particular those involving the R2 and R4″ positions, are important for biological activity. Since bulkier groups at position R2 are associated with a more beneficial effect on strains having a lax cell wall (Δerg6), they probably promote binding or adsorbing of the molecule on the cell wall. Moreover, if these groups are inferred to be more polarizable, the molecules are more beneficial to the wild-type strain than to deletion strains, probably because of a less efficient detoxification by the cell or due to a higher ability of the molecule to enter into the cell. Even more relevant is the chemical group present at position R4″. The groups at this position, being the most variable in the chemical library studied, clearly showed that several features have to be taken into account when trying to improve the activity of a certain molecule. The high deleterious effect induced on Δerg6 cells by compounds having high steric hindrance at R4″ can be ascribable to the fact that those molecules are able to permeate the cell wall because of the defect in the ergosterol biosynthesis pathway associated with this deletion. A secondary amide in this position makes the deleterious effect even more pronounced. Indeed, the fungicide compound with the highest activity of our chemical library had an ethylamine at R4″, and induced the highest rate of ODst reduction in all tested strains.

By analyzing the whole set of compounds, we were able to identify the chemical features (substituents or scaffolds) which are most responsible for the biological effect. We further show that results obtained with a thorough biological screen carried out on deletion strains (whose data were not used to train the models) highly overlap with those coming from a preliminary screen that was followed by stepwise regression. Despite suggesting to synthesize and test *in vivo* novel compounds was outside the scope of this work, we showed that the regression approach can help in guiding molecular testing on those molecules that are predicted to be more active. Thus, this simple strategy can be successfully exploited for the unbiased identification of a subset of most active constituents from the screening of very large compound libraries.

## Author contributions

MB and IS conceived and coordinated the study and wrote the paper. IS performed the analyses. AT synthesized the molecules, provided technical assistance and contributed in discussing the results. TW and DC contributed in discussing the results. The manuscript was written through contributions of all authors. All authors have given approval to the final version of the manuscript.

## Funding

IS was supported by a Fellowship from the Wellcome-Warwick Quantitative Biomedicine Programme.

### Conflict of interest statement

The authors declare that the research was conducted in the absence of any commercial or financial relationships that could be construed as a potential conflict of interest.

## Abbreviations

BTAa: Bicycles from Tartaric acid and Amino acids
ODst: Optical Density at the culture stationary phase
YPD: Yeast Peptone Dextrose
DMSO: DiMethyl SulfOxide
PCA: Principal Component Analysis
RDA: Redundancy Analysis
AIC: Akaike Information Criterion.
