# Supplementary material for: Relations between Effects and Structure of Small Bicyclic Molecules on the Complex Model System Saccharomyces cerevisiae
Source: Front Pharmacol. 2017 Mar 30;8:170. doi: 10.3389/fphar.2017.00170 (PMC5371657; doi:10.3389/fphar.2017.00170)
Supplement: Supplementary file 2 [file Supplementaryfigures.pdf]

Supplementary Material

Article Title

Relations Between Effects and Structure of Small bicyclic molecules on the Complex Model System *Saccharomyces cerevisiae*

Brilli M., Trabocchi A., Weil T., Cavalieri D., Stefanini I.

This PDF file includes:

Supplementary Figure 1: drawings of the tested molecules

Supplementary Figure 2: relationship among Scaffold A, B, C of the selected molecules and their activity on the tested yeast strains.

Supplementary Figure 3: relationship among BTK, BTG and BTF and their activity on the tested yeast strains.

Supplementary Figure 4: relationship among hindrance or polarizability and themolecule biological effects.

Supplementary Figure 5: The presence of an hydrogen or of a benzyle at position R2 changes the molecule effect on W303 cell growth.

Other Supplementary Materials for this manuscript includes the following:

Table S1: Details on the effects and chemical characteristics of the compounds composing the library

Table S2-S4: Wilcox test results (p-values) of the comparisons carried out among the effects induced by the selected molecules on the wild-type W303 strain.

Table S5: Stepwise regression analysis results. Details are in decreasing AIC (Akaike Information Criterion) value order. Light gray cells includes details of the best models (these having the Lowest AIC value). Symbols are listed in Supplementary materials.

Table S6: Stepwise regression analysis results, coefficients calculated for the best model.

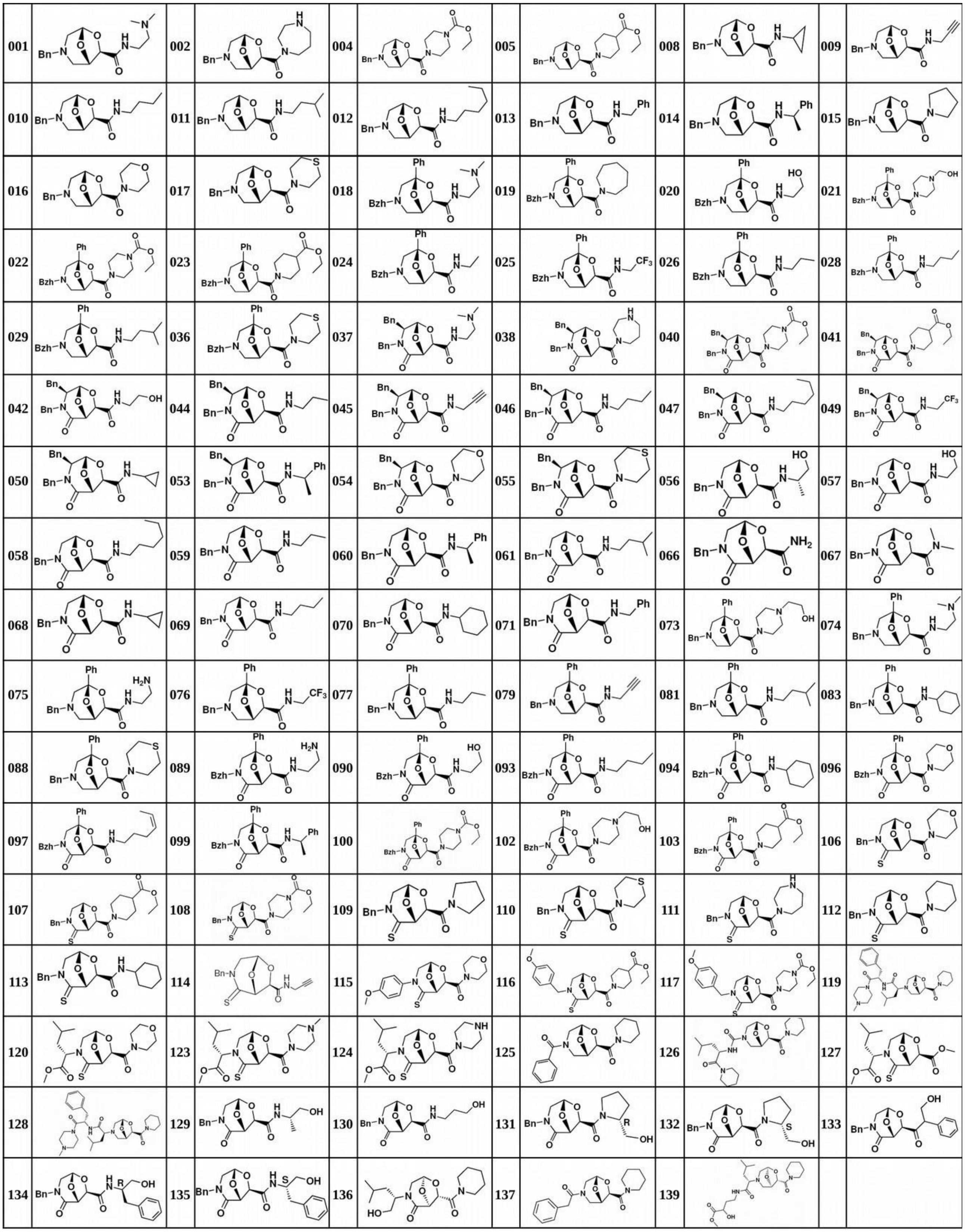

Supplementary Figure 1: drawings of the tested molecules

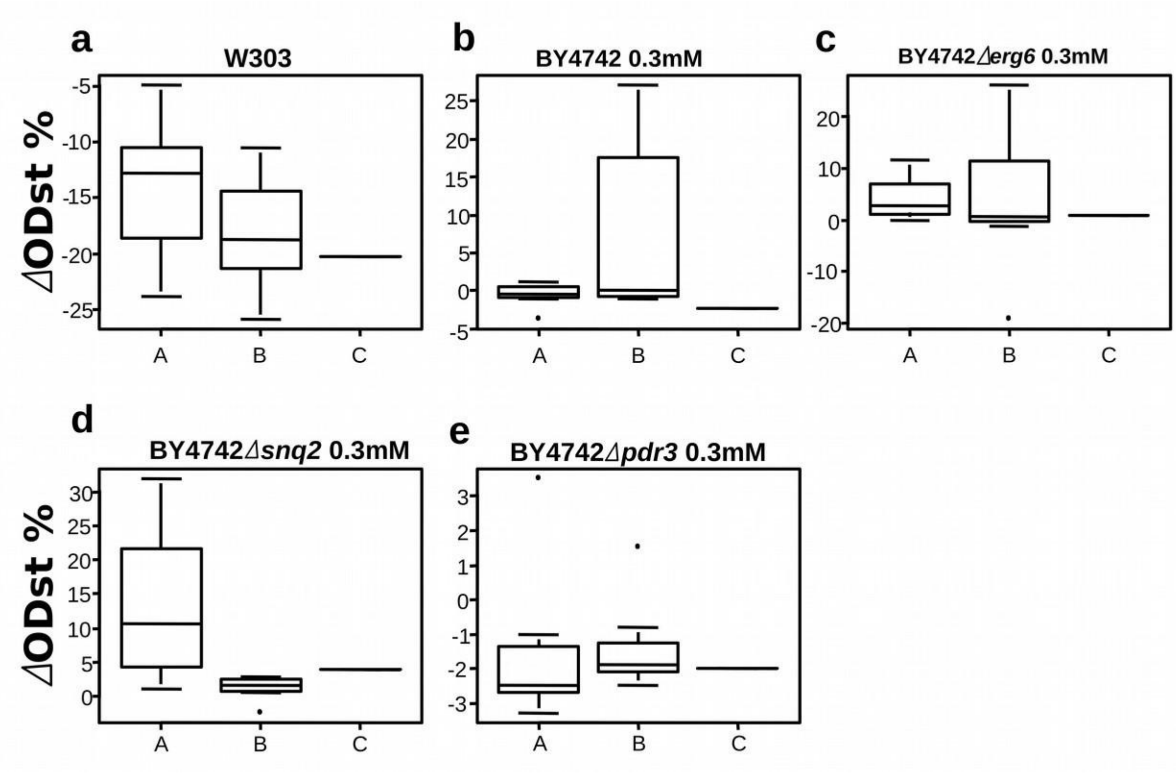

Supplementary figure 2: relationship among Scaffold A, B, C of the selected molecules and their activity on the tested yeast strains. a- Boxplot showing the effects on the wild-type W303 strain culture ODST caused by the molecules grouped accordingly to the scaffold classification showed in Fig. 1a; ***b-*** Boxplot showing the effects on the wild-type BY4742 strain culture ODST caused by the molecules grouped accordingly to the scaffold classification showed in Fig. 1a; ***c-*** Boxplot showing the effects on the BY4742*Δerg6* deletion strain culture ODST caused by the molecules grouped accordingly to the scaffold classification showed in Fig. 1a; ***d-*** Boxplot showing the effects on the BY4742*Δsnq2* deletion strain culture ODST caused by the molecules grouped accordingly to the scaffold classification showed in Fig. 1a; ***e-*** Boxplot showing the effects on the BY4742*Δpdr3* deletion strain culture ODST caused by the molecules grouped accordingly to the scaffold classification shown in Fig. 1a.

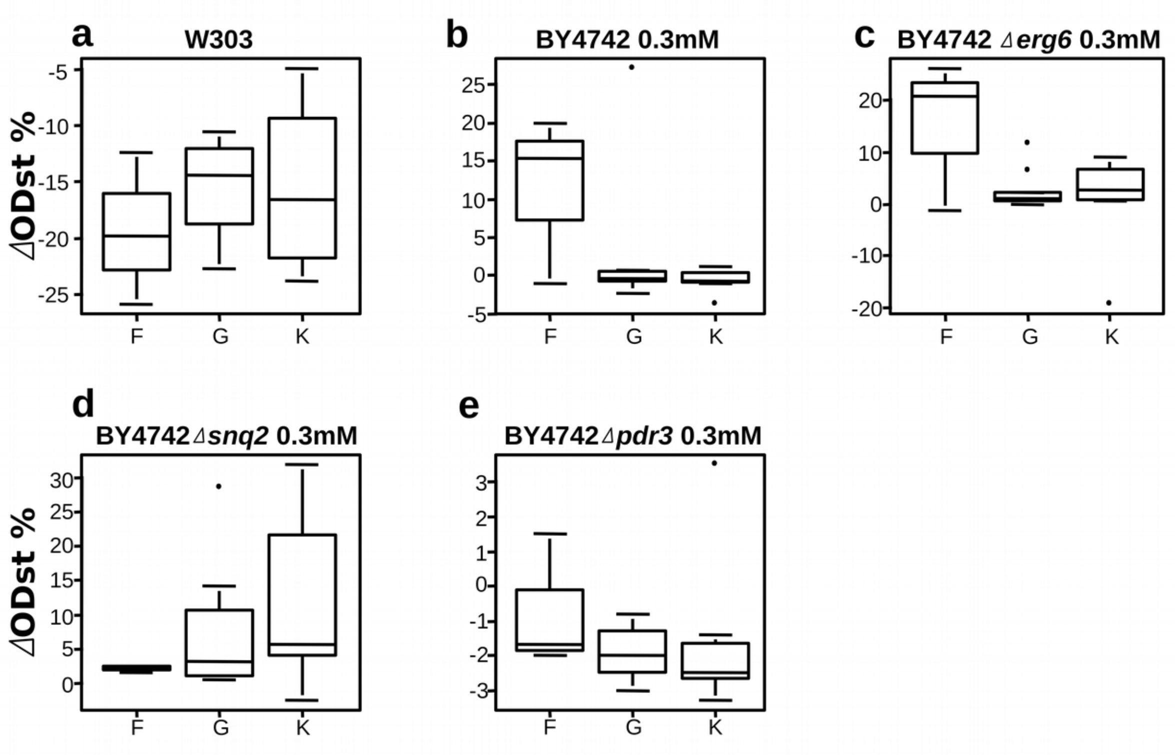

Supplementary figure 3: relationship among BTK, BTG and BTF and their activity on the tested yeast strains. a- Boxplot showing the effects on the wild-type W303 strain culture ODST caused by the molecules grouped accordingly to the scaffold classification showed in Fig. 1b; ***b-*** Boxplot showing the effects on the BY4742 strain culture ODST caused by the molecules grouped accordingly to the scaffold classification showed in Fig. 1b; ***c-*** Boxplot showing the effects on the BY4742*Δerg6* deletion strain culture ODST caused by the molecules grouped accordingly to the scaffold classification showed in Fig. 1B; ***d-*** Boxplot showing the effects on the BY4742*Δsnq2* deletion strain culture ODST caused by the molecules grouped accordingly to the scaffold classification showed in Fig. 1B; ***e-*** Boxplot showing the effects on the BY4742*Δpdr3* deletion strain culture ODST caused by the molecules grouped accordingly to the scaffold classification showed in Fig. 1b.

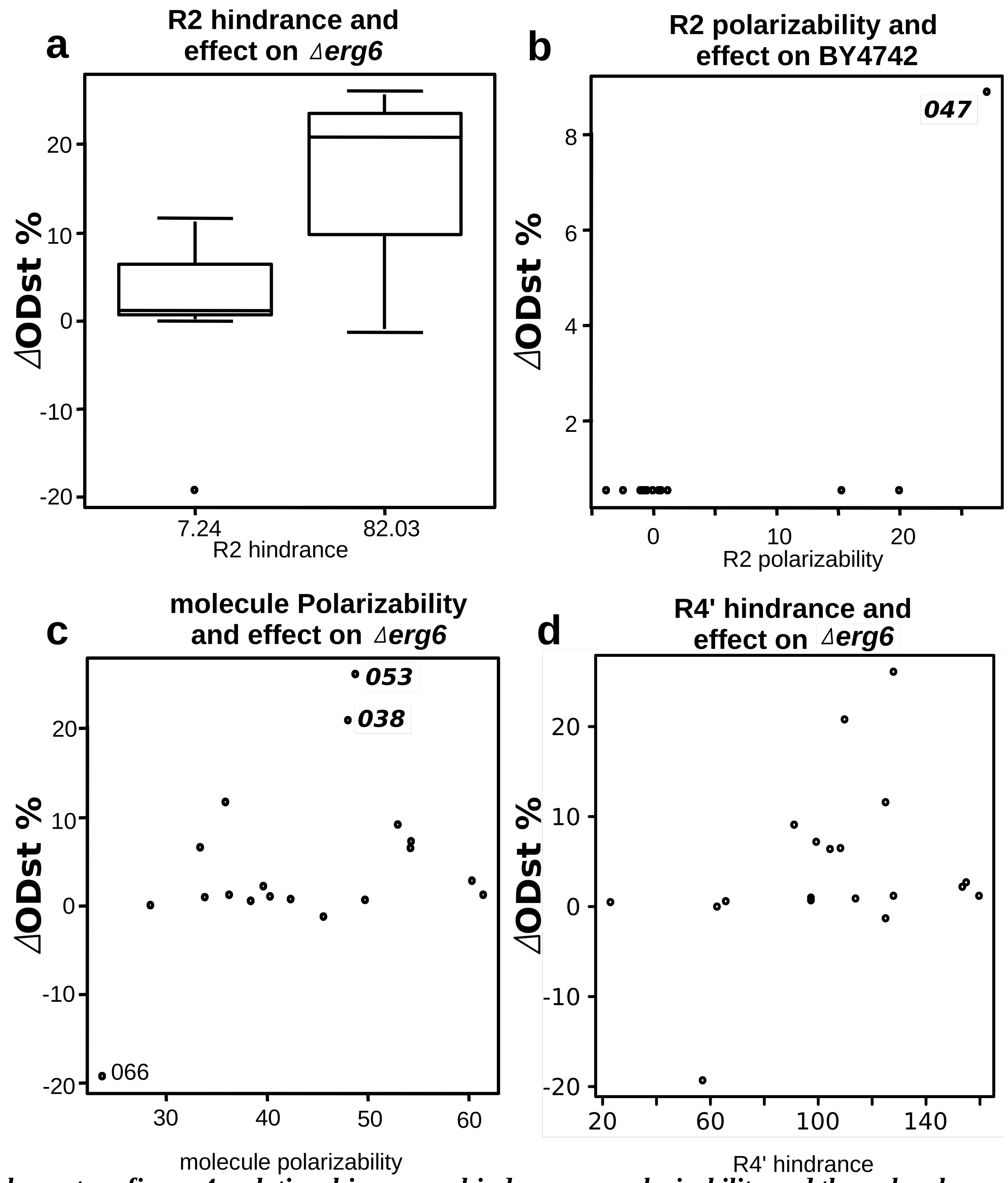

Supplementary figure 4: relationship among hindrance or polarizability and themolecule biological effects. a- Boxplot showing the effects on the BY4742*Δerg6* deletion strain culture ODST caused by the molecules grouped accordingly to the R2 residual steric hindrance; ***b-*** plot showing the effects on the wild-type BY4742 strain culture ODST caused by the molecules grouped accordingly to the R2 residual polarizability; ***c-*** plot showing the effects on the BY4742*Δerg6* deletion strain culture ODST caused by the molecules grouped accordingly to the molecule polarizability; ***d-*** plot showing the effects on the BY4742*Δerg6* deletion strain culture ODST caused by the molecules grouped accordingly to the R4’ steric hindrance.

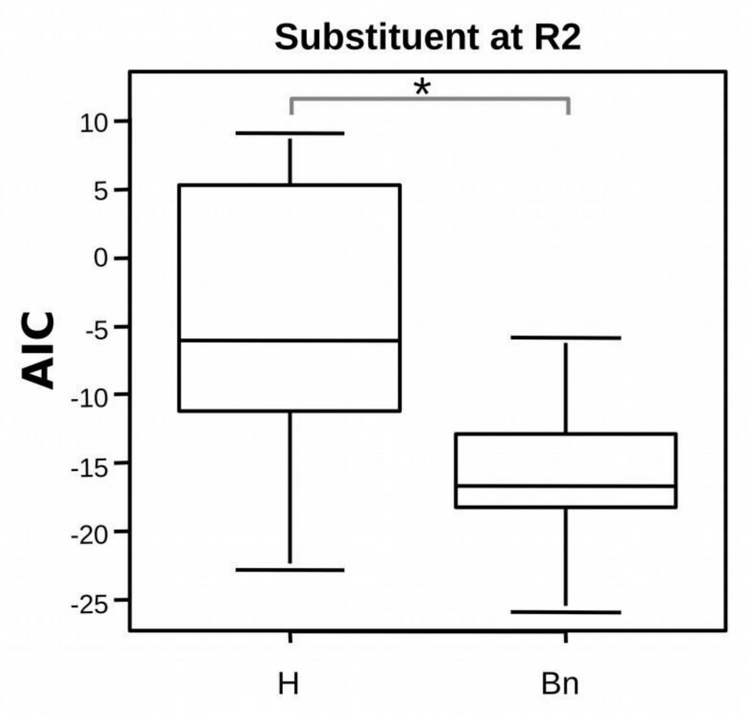

Supplementary Figure 5: The presence of an hydrogen or of a benzyl at position R2 changes the molecule effect on W303 cell growth. Data represent the subset of molecules inducing ΔOD>5 or <-5 bearing the substituents selected by mean of stepwise regression analysis. *p=0.01291 Wilcox test.
